# Improving the Estimation of Risk-Adjusted Grouped Hospital Standardized Mortality Ratios Using Cross-Jurisdictional Linked Administrative Data: A Retrospective Cohort Study

**DOI:** 10.3389/fpubh.2017.00013

**Published:** 2017-02-08

**Authors:** Katrina Spilsbury, Diana Rosman, Janine Alan, Anna M. Ferrante, James H. Boyd, James B. Semmens

**Affiliations:** ^1^Centre for Population Health Research, Curtin University, Perth, WA, Australia; ^2^Data Linkage, Department of Health WA, Perth, WA, Australia; ^3^PHRN Centre for Data Linkage, Centre for Population Health Research, Curtin University, Perth, WA, Australia

**Keywords:** cross-jurisdictional record linkage, hospital standardized mortality ratios, risk adjustment, epidemiology, cohort studies

## Abstract

**Background:**

Hospitals and death registries in Australia are operated under individual state government jurisdictions. Some state borders are located in heavily populated areas or are located near to major capital cities. Mortality indicators for hospital located near state borders may not be estimated accurately if patients are lost as they cross state borders. The aim of this study was to evaluate how cross-jurisdictional linkage of state hospital and death records across state borders may improve estimation of the hospital standardized mortality ratio (HSMR), a tool used in Australia as a hospital performance indicator.

**Method:**

Retrospective cohort study of 7.7 million hospital patients from July 2004 to June 2009. Inhospital deaths and deaths within 30 days of hospital discharge from four state jurisdictions were used to estimate the standardized mortality ratio of hospital groups defined by geography and type of hospital (grouped HSMR) under three record linkage scenarios, as follows: (1) cross-jurisdictional person-level linkage, (2) within-jurisdictional (state-based) person-level linkage, and (3) unlinked records. All public and private hospitals in New South Wales, Queensland, Western Australia, and public hospitals in South Australia were included in this study. Death registrations from all four states were obtained from state-based registries of births, deaths, and marriages.

**Results:**

Cross-jurisdictional linkage identified 11,116 cross-border hospital transfers of which 170 resulted in a cross-border inhospital death. An additional 496 cross-border deaths occurred within 30 days of hospital discharge. The inclusion of cross-jurisdictional person-level links to unlinked hospital records reduced the coefficient of variation among the grouped HSMRs from 0.19 to 0.15; the inclusion of 30-day deaths reduced the coefficient of variation further to 0.11. There were minor changes in grouped HSMRs between cross-jurisdictional and within-jurisdictional linkages, although the impact of cross-jurisdictional linkage increased when restricted to regions with high cross-border hospital use.

**Conclusion:**

Cross-jurisdictional linkage modified estimates of grouped HSMRs in hospital groups likely to receive a high proportion of cross-border users. Hospital identifiers will be required to confirm whether individual hospital performance indicators change.

## Introduction

Advances in information technology are changing the research environment in public health with increasing access to affordable, large, and complex administrative and surveillance health datasets. The potential of such data to improve population health outcomes is undisputed as whole populations can be followed more precisely in time and space. It has been proposed that precision public health could have particular benefit in preventative health with earlier detection and more precise risk estimates (1). However, the ethical and legal responsibility of protecting individual confidentiality must be balanced against the health benefits as these large amounts of data are brought together.

Following a $20 million government investment strategy, the Population Health Research Network (PHRN) was established to develop an accurate, reliable, and load-bearing national capability for data linkage in Australia. In 2009, the Centre for Data Linkage (CDL) was established within Curtin University and it provides the secure data linkage infrastructure necessary for cross-jurisdictional linkage of health-related data in Australia (2). The PHRN commissioned several proof of concept projects to demonstrate the feasibility and benefit of linking large datasets from across the country; the findings presented are from the first of these projects with the aim of demonstrating how estimation of the hospital standardized mortality ratio (HSMR) can be improved through cross-jurisdictional linkage.

Deaths in hospitals have long been of interest as an indicator of the quality of hospital care. The HSMR is an attempt to measure whether a hospital has a higher (or lower) number of hospital-related deaths relative to the overall mortality experience. HSMR is calculated by dividing the observed number of deaths by the expected number of deaths in that hospital. The expected number of deaths is estimated as the average of all deaths in all hospitals after accounting for case-mix variation by a range of possible risk-adjustment methodologies.

Hospital standardized mortality ratios as a measure of hospital quality of care have been the subject of considerable debate as to their value and how they should be used. It has been argued that HSMRs are a poor indicator of quality of care for several reasons. First, risk adjustment usually relies on variables collected from administrative data and not all may have been identified and reported accurately (3); second, a non-constant association of case-mix variables with death across hospitals could result in biases referred to as the constant risk fallacy (4), third, the statistical phenomenon that smaller hospitals are more likely to occur at the top and bottom of league tables (5), fourth, the fact that most hospital deaths are not avoidable means there is low signal to noise ratio in trying to assess the rarer preventable deaths (6); fifth, concerns have been raised that hospitals may modify their coding practices or policies, such as refusing to accept very ill patients in an attempt to modify their HSMR (7); and finally, there is very little consistent or reliable evidence that hospitals with higher HSMRs actually provide poorer quality of care (8, 9).

Proponents of the HSMR argue that they should be used as a screening tool that alerts institutions to a possible problem rather than being a definitive measure of quality of care (10). Moreover, they counter that HSMRs are computed from data already existing in hospital databases and therefore are practical and cost-efficient to estimate (11), the constant risk fallacy is unlikely to be an issue for most hospitals (12), they are only used as a small part of an overall system for monitoring quality of care (10) and they can be used to monitor hospital changes over time (11).

In Australia, the Australian Commission on Safety and Quality in Health Care (ACSQHC) developed a toolkit that contains a set of risk-adjusted coefficients constructed from national inhospital mortality data (13). This enables hospitals to compare their HSMR against the Australian average. While practical to implement, a limitation of the current Australian approach for estimating HSMRs is that they are based on unlinked hospital records. This means that (i) multiple hospital records belonging to the same individual may not be brought together even if they are part of the same hospital admission that will fail to describe the patient pathways accurately or account for patient transfer policies, (ii) any deaths that occur soon after hospital discharge are not captured and therefore the HSMR is subject to discharge biases, and (iii) important historical or longitudinal patient characteristics are not available for use in the case-mix risk-adjustment process.

In the absence of a unique person identifier in Australia, some of these limitations can be overcome by using person-level linkage methods. Until recently, person-level linkage of administrative hospital and death records has been limited to only two standalone state-based data linkage centers; the Western Australia (WA) Data Linkage System and the Centre for Health Record Linkage in New South Wales (NSW). A constraint of state based or within-jurisdictional person-linkage is that it cannot follow patients if they cross state borders to attend hospital, a problematic issue when major urban areas such as Brisbane (QLD) are located close to a heavily populated region across a state border (NSW). Cross-jurisdictional linkage can overcome this limitation.

Cross-jurisdictional linkages of hospital and death records from NSW, WA, SA, and Queensland (QLD) were generated by the CDL, the first study in Australia to combine hospital and death data from multiple jurisdictions at the person level (14). This allowed an understanding of the patterns of cross-border hospital use not previously attempted (15). It further enabled assessment of the impact of cross-jurisdictional person-level linkage on the estimation of HSMRs. Due to hospital confidentiality concerns, identification of individual hospitals was not possible for this proof of concept study; therefore, estimated standardized mortality ratios were limited to groups of hospitals based on peer group and geographical location instead, that is, a grouped hospital SMR (GHSMR).

## Materials and Methods

### Study Design

A retrospective cohort of all persons who were discharged (separated) from a NSW, WA, SA, or QLD participating hospital during the period 1st July 2004 to 30th June 2009 was identified. An additional 5 years of prior hospital separation records back to 1st July 1999 (where available) were used to identify past history of inpatient hospital use and preexisting comorbid medical conditions.

The main outcome measure was hospital-related deaths: both inhospital deaths and deaths that occurred within 30 days of separating from the last hospital stay. These deaths were used to estimate SMRs under three different record linkage scenarios, as follows: (1) cross-jurisdictional linkage, (2) jurisdictional (state-based) linkage, and (3) unlinked records. Ethical approval for this study was obtained from Human Research Ethics Committees in WA Health, QLD Health, SA Health Departments, the Cancer Institute NSW, and Curtin University (WA).

A detailed description of the hospital and death records used in this study have been published elsewhere (15). Briefly, inpatient records from public, psychiatric, and private hospitals, and private day surgery centers were available from NSW, WA, and QLD. SA provided public hospital inpatient records only. Death registration data were obtained from state-based registries of births, deaths, and marriages. The CDL created a set of person-level national linkage keys that linked all the hospital and death registration records across the four jurisdictions. These keys allowed the data custodians from each jurisdiction to provide relevant de-identified extractions of clinical and death data for analysis. The details of the cross-jurisdictional linkage process involved in this study are presented elsewhere (16).

### Data Cleaning and Standardization

Hospital records from the four jurisdictions underwent extensive cleaning and standardization to maximize analytical comparability. A standard set of exclusions included hospital boarders, organ procurements, aged care residents, funding hospital (duplicate) cases, canceled procedure admissions, unqualified newborns, and healthy qualified newborns. Records with missing age, sex, principal diagnosis or mode of separation were also excluded. Consensus categorical variables were constructed based on the variables from the jurisdictions that provided the least number of categories compared to other jurisdictions.

A number of jurisdictional coding differences were observed. For example, admissions for chemotherapy (ICD-10-AM code Z51.1) in public hospitals in NSW are mostly coded as outpatient events and were not included in the data, whereas they were coded as inpatient events and included in the data from the other three jurisdictions. Jurisdictional variations were identified by systematic cross-checking and with reference to the published metadata and local expertise.

### Variable Definitions

Eligible hospital stays had (i) acute care or, for multiple episodes of care, the first episode of care was acute care, (ii) a final discharge date that fell from 1st July 2004 to 30th June 2009, (iii) a total length of stay less than 1 year, and (iv) an Australian postcode of residence.

For this study, a hospital transfer was defined as a compilation of hospital records that indicated either a subsequent transfer to another acute hospital or a statistical discharge within the same hospital had occurred. A maximum of 48 h was allowed for a patient to transfer from one acute hospital to another.

The principal reasons for admission to hospital (principal diagnosis codes) were aggregated into broader diagnostic groups by recoding the ICD-10-AM code into one of 256 Clinical Classification System (CCS) groups (17). These 256 CCS groups were further aggregated into 150 CCS group classifications similar to that reported by Campbell et al. (18) when constructing the summary hospital mortality index (SHMI) with some modification. For example, there were sufficient numbers of hospital stays to create a separate category for melanoma and non-melanoma skin cancers.

The Quan ICD-10 coding algorithm for the Deyo/Charlson index was used to create a Charlson comorbidity score (19) with a 5-year look back period for person-level-linked records and no look back period for unlinked hospital records. An average depth of coding weighting was estimated to account for the extent to which preexisting medical conditions were coded in each calendar year and within each hospital group. Variation in the comprehensiveness of hospital coding practice has been shown to impact estimation of HSMRs (20).

### Risk Adjustment and GHSMR Estimation

Estimation of GHSMRs was restricted to (A) principal referral and specialist women’s and children’s hospitals, (B) large hospitals, (C) medium hospitals, and (D) small acute hospitals peer groups as defined by the Australian Institute of Health and Welfare (21). Hospital groups were created by splitting the four peer groups A, B, C, and D into smaller categories defined by geographical location and state jurisdiction; this created 43 different hospital groupings. Hospital geographic classifications were major city, inner regional, outer regional, and remote as assigned by the providing jurisdiction. Hospital-related deaths were attributed to the hospital associated with the first episode of care in a multicare episode hospital stay involving transfers.

The method for risk adjustment was based on that reported for the SHMI (18) with modification. The probability of a hospital-related death was estimated by fitting separate logistic regression models for each of the 48 most frequent CCS diagnostic groups that accounted for 80% of hospital-related deaths for each of the three different linkage scenarios. The dependent variables in these models were either (a) all hospital-related deaths (inhospital and 30-day deaths) or (b) inhospital deaths only. The independent variables used in these models were those factors likely to be associated with patient mortality outcomes and included patient age as quadratic term, gender, year, average depth of ICD coding weighting, length of stay, raw Charlson comorbidity score (5-year look back period), urgency of the hospital admission, accessibility to services (ARIA+), socioeconomc status (Index of Relative Social Disadvantage), marital status, aboriginality, number of times hospitalized in previous 5 years, whether the hospital stay involved intensive care or a ventilator, and whether the hospital stay involved a hospital transfer. Hospitalization history was excluded from the unlinked regression models. The discriminatory ability of each of these regression models to correctly classify hospital-related deaths was quantified using the area under the curve (c-statistic) from receiver-operating characteristic (ROC) analysis.

The expected number of hospital-related deaths was calculated by summing the probability of a hospital-related death for each hospital stay over each of the 43 different hospital groups. The GHSMRs were calculated as the ratio of actual observed number of hospital-related deaths in a hospital grouping to the expected number of deaths in that hospital grouping × 100. The 95% confidence intervals for the GHSMR estimates were calculated using Byar’s approximation to the exact results based on the Poisson distribution (22). To increase the sensitivity of detecting differences in GHSMRS between those estimated using cross-jurisdictional links and those estimated using jurisdictional links in the absence of unique hospital identifiers, a subset analysis was performed. This involved conducting the risk adjustment and GHSMR estimation on the subset of patients who lived in statistical local areas (SLAs) where more than 1,200 patients crossed a state border to attend hospital over the 5-year study, an effective sample size of 302,191 (2.7%) hospital stays. GHSMRs are presented only for the hospital groups with more than 10 observed deaths within this population subset.

## Results

There were 19.7 million hospital records from July 2004 to June 2009 that met the inclusion criteria. After applying jurisdictional person-level linkages that allowed multiple records pertaining to the same individual and admission to be bought together into a single hospital stay, the total number of records reduced 4% to 18.9 million hospital stays, which represented 7.8 million unique individuals (Table 1).

**Table 1:** **The number and percentage of hospital stays, episodes of care, individual patients, and hospital-related deaths in the four participating jurisdictions under the three different data linkage scenarios**.

	NSW^d^		WA		QLD		SA^c^		Total
	*N*	%	*N*	%	*N*	%	*N*	%	
1. Cross-jurisdictional linkage									
Hospital stays	8,723,879	46.2	2,799,646	14.8	5,919,025	31.4	1,427,780	7.6	18,870,330
Individuals	3,660,991	47.9	1,094,303	14.3	2,286,449	30.0	608,921	8.0	7,650,664
Inhospital deaths^a^	104,439	2.9	23,725	2.2	58,484	2.6	20,073	3.3	206,721
30-day deaths^b^	33,868	1.0	8,038	0.8	16,496	0.7	7,922	1.4	66,324
Hospital stays by non-residents^e^	157,851	1.8	11,834	0.4	155,620	2.6	27,664	1.9	352,969
Cross-border transfers sent	9,442	84.9	65	0.6	1,278	11.5	331	3.0	11,116
Cross-border transfers received	1,584	14.2	28	0.3	8,164	73.4	1,340	12.1	11,116
Cross-border deaths	239	48.2	12	2.4	205	41.3	40	8.1	496
**2. Within-jurisdictional linkage**									
Hospital stays	8,725,254	46.2	2,799,122	14.8	5,927,122	31.4	1,429,133	7.6	18,881,226
Individuals	3,699,822	47.7	1,104,067	14.2	2,331,133	30.2	617,175	8.0	7,762,197
Inhospital deaths^a^	103,958	2.81	23,719	2.15	58,760	2.51	20,113	3.26	206,550
30-day deaths^b^	33,666	0.9	8,030	0.7	16,292	0.7	7,903	1.3	65,891
**3. Unlinked separation-level data**									
Hospital records	9,130,886	46.4	2,881,774	14.7	6,165,476	31.4	1,479,786	7.5	19,657,922
Inhospital deaths	96,556	1.1	19,446	0.7	50,041	0.8	19,157	1.3	185,200

*^a^Percentage represents proportion of deaths in individuals who had a hospital stay in the 5-year period*.

*^b^Percentage represents proportion of 30-day deaths in individuals who were discharged alive from their last hospital stay (i.e., excluded individuals who died in hospital)*.

*^c^SA data included public hospitals only*.

*^d^NSW inpatient data include deaths in emergency departments*.

*^e^Proportion of hospital stays by non-state residents (cross-border users) relative to all hospital stays in the jurisdiction*.

The further addition of cross-jurisdictional linkages brought together both episodes of care that involved hospital transfers across a state border (*n* = 11,116) into a single hospital stay and allowed patients who had hospital stays in more than one jurisdiction to be merged into a single patient. Cross-jurisdictional linkage reduced the number of unique hospital stays by 0.6% and reduced the total number of individual patients by a further 1.4% compared with jurisdictional linkages.

The number and proportions of hospital-related deaths also varied depending on the data linkage scenario used (Table 1). When cross-jurisdictional linkage was used, there were 207,000 inhospital deaths identified, of which 48,380 (23%) occurred during hospital stays involving multiple episodes of care (transfers). Around 22,000 of these inhospital deaths were identified only in the person-linked data scenarios compared with unlinked data because the primary acute care episode of care in a hospital stay involving a transfer was linked to a subsequent non-acute episode of care in which the death occurred.

A further 170 inhospital deaths were identified using cross-jurisdictional linkage compared with jurisdictional links because it detected patients who had a hospital transfer across a state border to receive non-acute care and who then died in hospital. Additionally, there were 496 patients who died within 30 days of discharge and their death was registered in a different jurisdiction; 433 deaths in a different jurisdiction and 53 patients who had dual death registrations (all were dual registered in QLD and NSW).

The logistic regression models used to estimate the probability of hospital-related death in each of the 48 most frequent diagnostic groups had areas under the ROC curve (c-statistics) that ranged from 0.95 for the cardiac arrest and ventricular fibrillation to 0.70 for non-hypertensive congestive heart failure; a consistent finding for both the cross-jurisdictional and single-jurisdictional linked data. The ability of the logistic regression models to correctly classify inhospital deaths in the unlinked separation-level data varied from the person-linked hospital data with a maximum c-statistic of 0.95 for biliary tract disease and a lower 0.82 for cardiac arrest and ventricular fibrillation. The average c-statistic for the unlinked separation-level data for inhospital deaths was 0.84, slightly less than the average for person-linked data models at 0.85.

**Figure 1:**
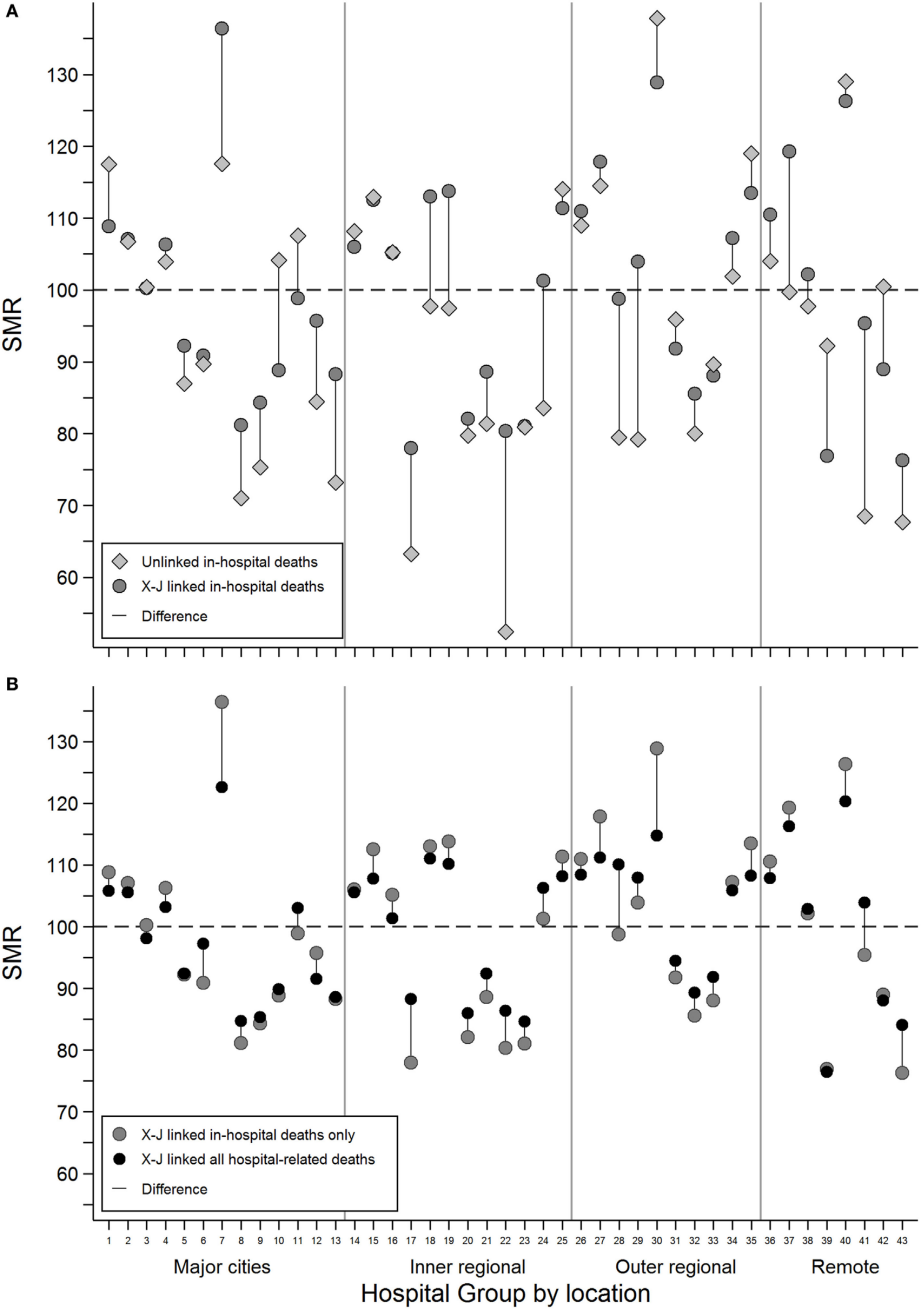
**The difference in estimated grouped hospital standardized mortality ratios between (A) unlinked inhospital deaths (gray diamonds) and cross-jurisdictional linked inhospital deaths (dark gray circles) and (B) cross-jurisdictional linked inhospital deaths (dark gray circles) and cross-jurisdictional linked all hospital-related deaths, inhospital, and 30-day deaths (black circles) for each of the 43 hospital groups defined by broad geographical areas and peer groups**.

Grouped hospital SMRs estimated using inhospital deaths only were compared for cross-jurisdictional and unlinked hospital records (Figure 1A). The addition of the person-level links allowed episodes of care for an individual to be bought together into a single admission and resulted in a change of GHSMR toward the group average GHSMR of 100 in most cases.

For example, Hospital Group 1 with a SMR of 118 (95% CI: 116–119) using unlinked data dropped to 109 (95% CI: 108–110) with person-level cross-jurisdictional linked records. For some hospital groups with relatively low numbers of observed deaths, the observed changes in GHSMR were not always statistically significant. For example, Hospital Group 7 with around 50 observed deaths had an unlinked GHSMR of 118 (95% CI: 83–162) that increased to 136 (95% CI: 101–180) with person-level cross-jurisdictional linkage.

The inclusion of deaths within 30 days of hospital discharge into the GHSMR estimates for the cross-jurisdictional linkage scenario resulted in GHSMR changes more consistently toward the group average (Figure 1B). In some cases, the addition of 30-day deaths reversed the change in GHSMR observed when person-level cross-jurisdictional links were first added to unlinked data (see Hospital Group 7 in Figures 1A,B for example). Overall, the inclusion of cross-jurisdictional person-level links to unlinked separation data reduced the coefficient of variation among the hospital groups from 0.19 to 0.15; the inclusion of 30-day deaths reduced the coefficient of variation further to 0.11.

**Figure 2:**
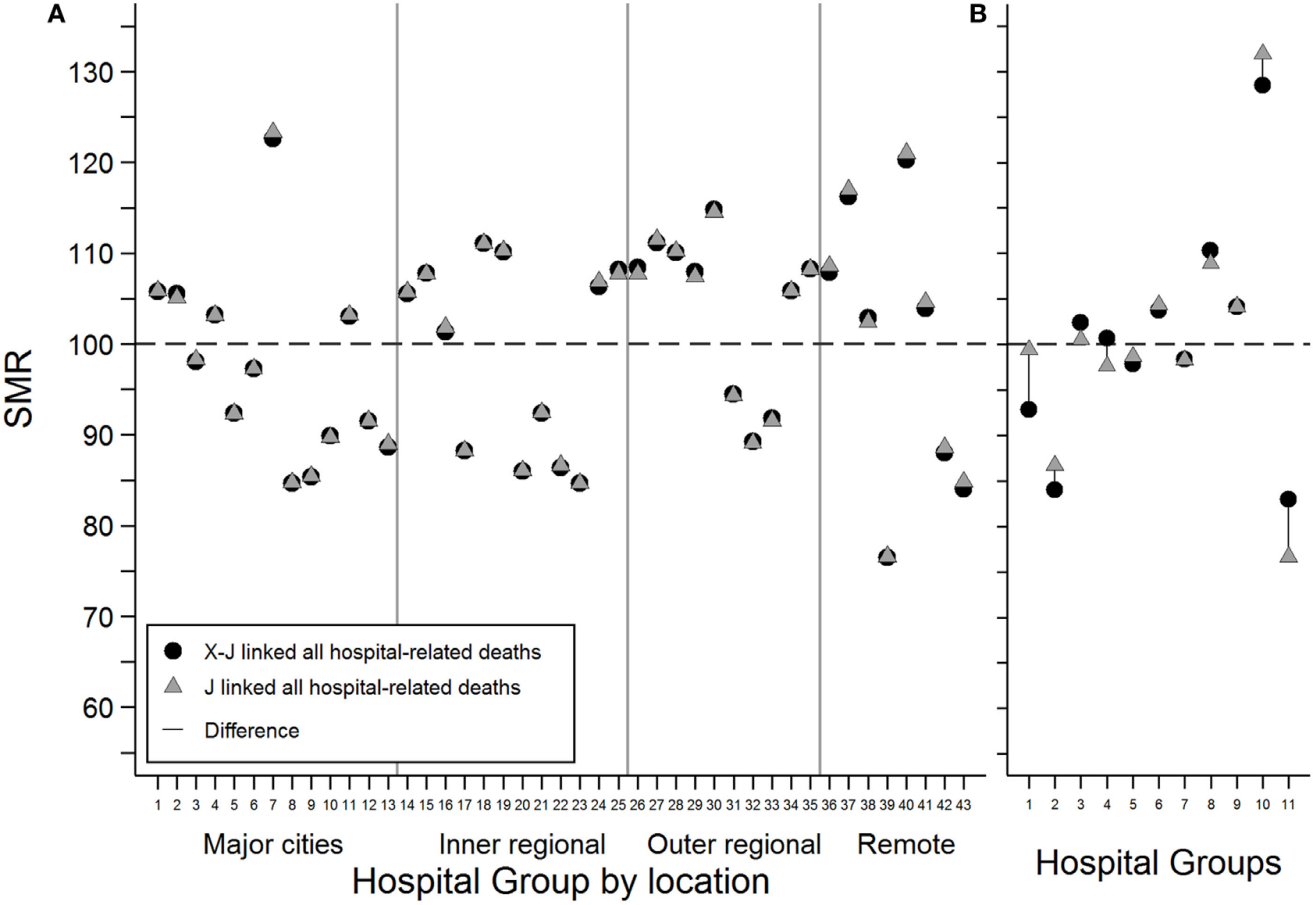
**The difference in estimated grouped hospital standardized mortality ratios for all hospital-related deaths between cross-jurisdictional linked (black circles) and jurisdictional linked (gray triangles) hospital records for (A) all hospital stays and (B) a subset of hospital stays restricted to patients living in statistical local areas with relatively high proportions of cross-border hospital users**. Only hospital groups with more than 10 observed deaths were included.

There were only minor changes to the GHSMR estimates when cross-jurisdictional linkages were compared to jurisdictional linkages (Figure 2A). Hospital groups in remote areas tended to show the greatest difference as a result of cross-jurisdictional linkage. To increase the sensitivity of this comparison due to the limitation of not having individual hospital identifiers, the GHSMR estimation was restricted to the subset of patients residing in SLAs with high proportions of cross-border hospital users (Figure 2B). This restriction demonstrated increased variation in GHSMRs estimated using cross-jurisdictional links compared with jurisdictional links for several of the 11 hospital groups that had more than 10 observed deaths.

## Discussion

We have demonstrated that using cross-jurisdictional linked hospital and death records can modify estimates of SMRs based on broad hospital groupings compared with both unlinked and jurisdictional linked records. For this study, the largest changes in GHSMRs for inhospital deaths were between unlinked records and person-level linked data. Person-level data allowed multiple episodes of care to be bought together into a single hospital stay. This allowed more accurate estimation of the number of patients, and their care pathways, and improved the identification of hospital-related deaths during non-acute care that were linked to an acute care admission. Additionally, the more complete ascertainment of patient comorbidity and hospital stay history improved the GHSMR estimation.

Including all 30-day deaths into the GHSMR estimation reduced the overall spread of GHSMRs and tended to bring outlying hospital groups toward the group average. This is consistent with previous work for NSW hospital data that showed that including 30-day deaths reduced the variation in HSMRs (23). It is likely that this overall reduction in variation occurs because including 30-day deaths into GHSMR estimation reduces the hospital-related death variation associated with early-discharge practices and varying hospital transfer processes.

Estimation of GHSMRs for hospital-related deaths using cross-jurisdictional links compared with jurisdictional links included additional deaths associated with the 11,116 cross-jurisdictional hospital transfers and the 496 cross-border hospital deaths. These additional deaths made only minor changes to the GHSMRs in this study because of the reduced sensitivity of using hospital groups rather than individual hospitals. In this study, individual hospital identifiers were not available and SMR estimation was restricted to broad geographical and peer group categories. It is expected that significant changes in mortality rates could result for hospitals located close to jurisdictional borders when cross-jurisdictional linkages are included at an individual hospital level. This hypothesis is supported by the larger effect observed for cross-jurisdictional linked GHSMRs when restricted to patients living in high cross-border hospital use regions.

The risk-adjustment method used in this report were designed to make full use of the linked data available and thus differs from the method presented in the toolkit developed by the ACSQHC for hospitals to estimate their HSMR core hospital-based outcome indicators (13). While the regression models used to estimate the expected number of hospital-related deaths had high c-statistics, the approach used here would be impractical to implement on a real-time basis for monitoring hospital performance unless timely access to death registration data to identify deaths within 30 days of discharge can be contrived.

A condition of data release for this study prevented identification of individual hospitals, which was a major limitation. This restriction was primarily the result of privacy concerns and prevented the comparison of individual hospitals with similar characteristics. As a result, the GHSMRs reported here cannot, nor are meant to be, interpreted in any clinically meaningful way. This limitation highlights that there are still ethical, legal, and social barriers to overcome before cross-jurisdictional linkage is implemented regularly in Australia. Ensuring public confidence in the technology of data linkage to maintain individual confidentiality, advocating for changes to out-dated legislation and providing a strong ethical base to research training undertaken by organization such as the PHRN and the Centre for Big Data Research in Health will contribute to positive change. Other innovations such as secure remote-access computer environments and the development and use of privacy-preserving record linkage techniques will continue to play a role in the future of data linkage.

## Conclusion

We have shown that linking individuals and their hospital stays across jurisdictional borders can modify estimates of standardized mortality ratios. Hospital identifiers will be required to confirm these findings. Improving the precision of the HSMR as a hospital performance indicator is particularly relevant for hospitals that are located close to borders or that have relatively high numbers of interstate travelers.

## Author Contributions

KS carried out the data manipulation, data analysis, and drafted the manuscript. DR conceived the design of the study, negotiated data acquisition, and contributed to manuscript preparation. JA conceived the design of the study, negotiated data acquisition, and contributed to manuscript preparation. AF contributed to data acquisition, data analysis, and manuscript preparation. JB contributed to data acquisition, data analysis, and manuscript preparation. JS contributed to overall study concept and critically reviewed the manuscript.

## Conflict of Interest Statement

KS, AF, and JB were funded by the PHRN to conduct the study reported here. The other authors declare no conflict of interest.
